# “What will you do after?”: Lessons from Academia and the World Beyond

**DOI:** 10.1177/17470218241236144

**Published:** 2024-03-07

**Authors:** Christopher R Madan

**Affiliations:** School of Psychology, University of Nottingham, Nottingham, UK

**Keywords:** Career paths, doctoral training, academic-adjacent roles, skill-transfer careers, career transition, psychology graduate careers

## Abstract

Determining post-PhD career options is a challenge for many Psychology PhD graduates. Here I provide a comprehensive overview of the diverse career trajectories available to graduates, drawing from interviews with 53 PhD graduates conducted as part of the two-volume *Academia and the World Beyond* book series. From these, I conducted a hierarchical qualitative classification to categorise and characterise potential career paths. The findings reveal a spectrum of opportunities, from traditional academic roles to “academic adjacent” and “skill-transfer” careers. This work underscores the versatility of Psychology doctoral training, providing skills that can support a wide array of career possibilities. The results serve as a guide for current and prospective PhD students—and their mentors—emphasising the variety of professional contexts where doctoral training is beneficial.

## Introduction

In the contemporary academic landscape, the journey from doctoral studies to a fulfilling career is often fraught with uncertainty. “What will you do after?” is not just a casual question posed by friends and family to Psychology PhD students; it’s a reflection of the broader challenge that these graduates face in navigating the complex terrain of career opportunities. Although academia has traditionally been the sought-after path, the limited availability of academic positions, coupled with the evolving dynamics of the job market (Afonja et al., 2021; Edge & Munro, 2015; Feibelman, 2011; Kelsky, 2015; Linder et al., 2020; Schillebeeckx et al., 2013), has necessitated a broader view of potential post-PhD career trajectories.

The significance of this challenge cannot be understated. For many PhD graduates, the transition from academia to the professional world is not just about securing a job—it’s about finding a role that aligns with their skills, passions, and the years of specialised training they’ve undergone. Moreover, with the diversification of industries and the increasing value placed on interdisciplinary skills, there’s a pressing need to understand how the competencies acquired during doctoral studies can be applied in various professional contexts (Bernery et al., 2022; Robinson & Nolis, 2020; Sinche et al., 2017). PhD graduates know that a wide range of nonacademic career paths exist, but the specifics are nebulous.

Here, I aim to shed light on this issue. Having interviewed 53 PhD graduates in a two-volume book series, *Academia and the World Beyond* (Madan, 2022a, 2024a), here I seek to provide an overview of the diverse career paths available to Psychology PhD graduates. Although individual narratives offer rich insights, there’s a need for a higher-level summary—a structured framework that categorises and characterises the broader career trajectories, be it in academia, roles that are “academic adjacent,” or those that predominantly rely on “skill transfer” of general doctoral skills, such as data science or project management (Madan, 2024b).

I conducted a hierarchical qualitative classification of the interviews to determine a more granular characterisation of post-PhD career paths relevant to Psychology PhD graduates. This classification was conducted based on a framework analysis, a methodological approach that allows for the systematic identification and interpretation of key themes and patterns (Gale et al., 2013; Parkinson et al., 2016; Ritchie & Spencer, 1994). This method enabled us to discern both the similarities and differences in the career journeys of the interviewees. By doing so, I hope to provide not just a descriptive account but a guide—a tool that can help current and future PhD students make informed decisions about their careers, grounded in real-world examples and a deep understanding of the transferability of their skills.

## Methods

### Participants

A total of 53 people were interviewed for *Academia and the World Beyond* Volumes 1 and 2 (Madan, 2022a, 2024a). All interviewees previously completed a PhD, most in Psychology or Neuroscience. Of the 22 interviewees from Volume 1, 10 were in academic positions and 12 in nonacademic positions. Of the 31 interviewees from Volume 2, all were in nonacademic positions—though these are predominately still what could be considered “academic-adjacent” career paths (Madan, 2024b). Some interviewees were introduced to the researcher by a previous interviewee (i.e., snowball sampling). All interviewees consented for their interviews to be published and shared as a chapter in their respective edited volume.

Of the 53 interviewees, 20 completed their PhDs in the United Kingdom, 14 in the United States, 9 in Canada, and 3 in Germany. Of the remaining seven interviewees, one each completed their PhDs in France, Ireland, the Netherlands, Finland, Sweden, Australia, and Brazil.

### Interview procedure

Interviewees were contacted directly and invited to be interviewed, with the interview published as a chapter in *Academia and the World Beyond*. Interviews occurred asynchronously via a shared cloud-based writing document (Google Docs) over the course of a week to a year, a few questions at a time, based on interviewee availability. Further details about the interview questions and methods are reported in Madan (2022b, 2024b).

### Data analysis

**Figure 1.**
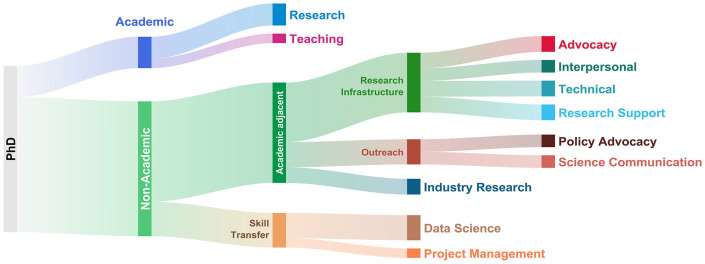
Hierarchical representation of post-PhD career path categories. Path width is proportional to the 53 individuals interviewed, *not* the relative frequency of career path within the job sector as a whole. For example, academic research is overrepresented. Terminal sets (i.e., groups with text labels on the right) represent 3–8 interviewees each.

Framework analysis involves five stages: familiarisation, identifying a framework, indexing, charting, and mapping and interpretation (Ritchie & Spencer, 1994; also see Parkinson et al., 2016). The researcher was already familiar with the interviewees as the interviews had been conducted over weeks (i.e., not in a single session), but portions were revisited throughout the process. The primary goal here was to cluster interviewees based on their career roles, with the intention of having hierarchical groups, where the terminal sets (i.e., smallest groups) were 3 to 7 interviewees. For all 53 interviewees, interviewee name, job title and employer, and some notes from the interview were written on flashcards. Interviewees were grouped for initial similarities, for example, science communication, industry research, or working in data-science roles. Charting involved determining relationships between smaller groups, for instance, that some roles were focused on supporting the training of future PhD students, community building, or developing technical solutions that support others’ research. Mapping and interpreting involved building the final hierarchical representation (shown in Figure 1) and reflecting on it for consistency. For each grouping, a justification had to be possible for why some interviewees were grouped together and others viewed as distinct. Some interviewees would weakly fit in another cluster and weaker relationships and distinctions are discussed in the Results section.

Two terminal sets ended with eight individuals (i.e., greater than planned 3–7), the academic research group and the data science group. Academic research could be separated into a separate set for academic management (e.g., department chair, head of research centre), but this is less relevant as a career target for someone currently completing a PhD. Data science roles appeared comparably varied to not warrant further categorisation.

## Results

The final hierarchical representation of post-PhD career paths is shown in Figure 1.

### Academic careers

Academic careers are centred on research, teaching, and service obligations. Research responsibilities involve producing original research and publishing papers. Teaching responsibilities include lecturing, grading, and advising students. Service includes committees, professional organisations, and reviewing. There is a high level of specialisation, flexibility in scheduling, and the ability to work independently. However, there is also instability due to short-term contracts, high competition for funding and positions, and unclear career advancement.

*Research*-focused academics tended to highlight the intellectual freedom and ability to pursue novel research questions as motivating factors in their career choice. They enjoyed analysing data, problem solving, and contributing new knowledge. These individuals suggested that students interested in research careers should gain experience in different lab settings and methodologies. Developing technical skills and learning to collaborate effectively were highlighted as important.

*Teaching*-focused academics were drawn to mentoring students, shaping curriculum, and communicating complex ideas. They found fulfilment in teaching itself rather than producing novel research. These interviewees emphasised the importance of gaining teaching experience through teaching associate positions or guest lecturing. Strong communication and interpersonal skills were highlighted.

Although both groups work in academia, their day-to-day experiences differ. Research-focused academics spend more time analysing data, writing grants and papers, and guiding students doing hands-on research. Teaching faculty have more direct student contact through classes, office hours, and advising. Research faculty focus more on specialised technical expertise in their field, while teaching faculty need broader knowledge to instruct and support students across a wider range of topics.

### Nonacademic careers

Nonacademic careers encompass a diverse set of roles across industry, government, nonprofits, and more. These roles tend to have greater structure, clear objectives, and better job security. These careers can offer opportunities to apply expertise to real-world problems with more direct impact than would be possible in academia. Many nonacademic careers offer better work–life balance, pay, and benefits relative to academia. However, there is less ability to freely choose projects or topics of interest.

Doctoral training can be described as involving three sets of skills: topic, methods, and general (Madan, 2024b). Those that are using their topic and/or method specific skills, aspects that are more unique to their PhD, can be characterised as being in “academic-adjacent” roles. Those using more general skills, such as data science or project management, are “skill-transfer careers.”

### Different paths within academic-adjacent careers

Research does not only happen because of academics working at universities. People need to work at funding agencies, develop technologies that facilitate research, and share research with the general public. Here, I characterised academic-adjacent careers as three different sets: research infrastructure, outreach, and industry research.

Research infrastructure involves supporting research but not leading it directly. This group is further composed of four subsets, discussed in more detail in the next section. Outreach career roles use research findings to create tangible change, either through policy or public understanding—this group has two subsets: policy advocacy and science communication. To contrast these two paths more explicitly—research infrastructure supports academia more internally; outreach focuses on communicating research external to academia, such as to policymakers or the general public.

Industry research involves applying PhD-related expertise to real-world problems and seeing their work translate into products or services. Their day-to-day work involved meetings, data analysis, writing, and presentations—like in academia. But industry research also included activities like prototype development, clinical trials, and advising clients. While not completely free to choose their own projects, they had input into research directions and opportunities to explore additional interests. These industry roles allowed them to continue pursuing topics they cared about, similar to academia. However, they felt their work could have greater societal impact through commercialisation.

### Exploring the research infrastructure career paths

Those in research infrastructure career paths were in roles that directly supported and facilitated academic research. In many cases, they worked “behind the scenes,” allowing academics and PhD students to focus on research. As with academic-adjacent careers more generally, these roles allow the individual to stay as part of the broader research community and continue to use their PhD-related expertise. There were four categories within this set of paths: advocacy, interpersonal, technical, research support.

*Advocacy* roles involve policy, funding, and promoting disciplines. Individuals in these roles expressed a desire to have broader impacts, beyond a specific topic of research. These individuals worked for funding agencies or academic societies.

*Interpersonal* roles focus on coordination and direct researcher support through training programmes and university support for PhD students. Some of these individuals expressed getting greater enjoyment from supporting other researchers and seeing them grow, than in doing research themselves. These individuals worked for nonprofit organisations that supported research communities or at universities in supporting PhD student skill development (e.g., “Graduate School” or “Researcher Academy” departments).

*Technical* roles apply specialised expertise to develop research infrastructure tools, platforms, and methods. These roles are more similar to what is sometimes considered a “research software engineer.” These individuals worked for companies that developed research tools—such as for implementing experiment programmes or data analysis, online platforms for recruiting participants, or companies that facilitate data sharing.

*Research support* roles assist research through equipment, workshops, or editing services. Some individuals have started their own business where they develop online training courses or in-person workshops related to their PhD methods skills. Others work at companies that develop scientific equipment (e.g., eye-tracking, psychophysiology, or brain imaging measurements) and work in sales or user training.

### Exploring the outreach career paths

Those in outreach career paths want research to make an impact. There are two groups here: policy advocacy and science communication.

*Policy advocacy* roles focus on directly informing government decision-making and are drawn to policy issues. These individuals use research to develop evidence-based policy recommendations.

Although there is some overlap with the advocacy group included as part of research infrastructure, the goals are somewhat different. Policy advocates interact heavily with government, politics, and legislation; research infrastructure advocacy roles (funders and academic societies) are more focused on grant management and community networking.

*Science communication* roles aim to increase public understanding and engagement, using social media to traditional media. Here, the target audiences are the general public. These individuals enjoy translating complex concepts for broad audiences.

### Skill-transfer careers

These careers use broader PhD competencies in work settings farther from academia. These more general skills gained during doctoral training include data science, project management, and critical thinking, rather than direct research topic or method knowledge. Motivations for moving to these careers tend to be focused on better work-life balance, compensation, and career stability—rather than remaining connected to a research field. This set of career paths is particularly associated with a feeling of loss of identity/grieving leaving academia, but individuals ultimately find satisfaction applying their skills in new contexts. Here, positions are particularly varied, including consulting, project management, data science, and require proactively developing industry-relevant abilities.

*Data science* roles focus more on technical skills like programming, computational modelling, and data analysis. *Project management* roles involve coordinating teams, managing budgets, and overseeing project timelines and outcomes. Both allow transferring general research abilities like critical thinking, communication, and attention to detail. Moreover, both provide opportunities to use skills in new contexts, with more visible career progression, and—for many—offer better work-life balance and pay compared with academia.

## Discussion

The diverse career trajectories of Psychology PhD graduates, as overviewed in this study, underscore the multifaceted nature of skills and competencies acquired during doctoral training. Although the traditional dichotomy of academic versus nonacademic is retained, the plethora of nonacademic career paths is enumerated and is no longer nebulous. Through a framework analysis, here I have mapped out these post-PhD career possibilities to reveal a landscape rich with opportunities, challenges, and potential for impact.

The key finding here is the specific subsets within the broad categories of nonacademic careers. For instance, within the overarching group of research infrastructure, there still remain several major paths that can now be more clearly understood, evaluated, and sought for. Zooming out, while academic roles predominantly leverage domain-specific knowledge, the concept of academic-adjacent roles highlights the versatility of doctoral training. These roles, which span research infrastructure, outreach, and industry research, underscore the value of both domain expertise and broader research skills in nonacademic settings. However, skill-transfer careers emphasise the adaptability of general doctoral competencies, such as data analysis, critical thinking, and project management, in diverse industries.

Several interviewees have moved between academia and nonacademic jobs. This could be going to a nonacademic role for a few years and then returning to academia—or continuing in academia until reaching assistant professor and then leaving. It is not necessarily one or the other as a career, but an ongoing—and winding—career journey. Many circumstances factor into career decisions, particularly personal values related to caring responsibilities and other geographic constraints, but also the job opportunities that arise within the relevant time periods.

This study provides a broad overview of potential career paths for Psychology PhD graduates, further insights can be drawn from the interviews themselves—published in full in the two-volume book series, *Academia and the World Beyond* (Madan, 2022a, 2024a). Although I had contacted the 53 individuals interviewed in these two volumes, there were others who I also contacted but were not interviewed (an additional 56 individuals), typically either due to time commitments or continued career transitions. (N.B. interviewing for the first volume occurred in the height of the COVID-19 pandemic.) However, even those not available to be interviewed would fit within the identified set of post-PhD career paths.

For current and prospective PhD students, this study serves as a guide to post-PhD possibilities. It’s essential to approach career decisions with an open mind, recognising the versatility and adaptability of doctoral training. Engaging in informational interviews, seeking mentorship outside of academia, and participating in internships or short-term projects can provide valuable insights and experiences, aiding in informed career decision-making. Findings also suggest that doctoral programmes might benefit from incorporating training opportunities that emphasise both domain-specific and general research skills (Madan, 2021, 2024b). By doing so, graduates would be better equipped to navigate the diverse career landscape, whether they choose to remain in academia, venture into academic-adjacent roles, or transition to skill-transfer careers. However, a PhD is not a commitment to academia—by being informed of options, PhD graduates can carve out meaningful and impactful career paths that align with their skills, passions, and aspirations.
